# Supplementary material for: Pyruvate Dehydrogenase Kinase 1 inhibition mediated oxidative phosphorylation enhancement in cartilage promotes osteoarthritis progression
Source: BMC Musculoskelet Disord. 2023 Jul 20;24:597. doi: 10.1186/s12891-023-06585-6 (PMC10357736; doi:10.1186/s12891-023-06585-6)
Supplement: Supplementary file 1 — Additional file 1. [file 12891_2023_6585_MOESM1_ESM.zip › KEGG.Gsea.1653622432464/KEGG_TYPE_I_DIABETES_MELLITUS.html]

Details for gene set KEGG\_TYPE\_I\_DIABETES\_MELLITUS[GSEA]

|  || Dataset | 55457\_collapsed\_to\_symbols.cls.cls #OA\_versus\_normal.cls.cls #OA\_versus\_normal\_repos |
| Phenotype | cls.cls#OA\_versus\_normal\_repos |
| Upregulated in class | normal |
| GeneSet | KEGG\_TYPE\_I\_DIABETES\_MELLITUS |
| Enrichment Score (ES) | -0.24511412 |
| Normalized Enrichment Score (NES) | -0.8823383 |
| Nominal p-value | 0.6877193 |
| FDR q-value | 0.8528661 |
| FWER p-Value | 1.0 |
Table: GSEA Results Summary

  

Fig 1: Enrichment plot: KEGG\_TYPE\_I\_DIABETES\_MELLITUS      
 Profile of the Running ES Score & Positions of GeneSet Members on the Rank Ordered List

  

| SYMBOL | TITLE | RANK IN GENE LIST | RANK METRIC SCORE | RUNNING ES | CORE ENRICHMENT || 1 | HLA-DMB | "major histocompatibility complex, class II, DM beta [Source:HGNC Symbol;Acc:HGNC:4935]" | 679 | 0.598 | -0.0008 | No |
| 2 | CD86 | CD86 molecule [Source:HGNC Symbol;Acc:HGNC:1705] | 689 | 0.596 | 0.0502 | No |
| 3 | HLA-DQA1 | "major histocompatibility complex, class II, DQ alpha 1 [Source:HGNC Symbol;Acc:HGNC:4942]" | 1334 | 0.452 | 0.0394 | No |
| 4 | HLA-DMA | "major histocompatibility complex, class II, DM alpha [Source:HGNC Symbol;Acc:HGNC:4934]" | 1769 | 0.388 | 0.0394 | No |
| 5 | CPE | carboxypeptidase E [Source:HGNC Symbol;Acc:HGNC:2303] | 1796 | 0.385 | 0.0708 | No |
| 6 | PTPRN2 | protein tyrosine phosphatase receptor type N2 [Source:HGNC Symbol;Acc:HGNC:9677] | 1965 | 0.365 | 0.0894 | No |
| 7 | TNF | tumor necrosis factor [Source:HGNC Symbol;Acc:HGNC:11892] | 2098 | 0.350 | 0.1095 | No |
| 8 | HLA-DOB | "major histocompatibility complex, class II, DO beta [Source:HGNC Symbol;Acc:HGNC:4937]" | 2147 | 0.344 | 0.1357 | No |
| 9 | ICA1 | islet cell autoantigen 1 [Source:HGNC Symbol;Acc:HGNC:5343] | 2253 | 0.332 | 0.1564 | No |
| 10 | GAD1 | glutamate decarboxylase 1 [Source:HGNC Symbol;Acc:HGNC:4092] | 2534 | 0.299 | 0.1606 | No |
| 11 | HLA-DQB1 | "major histocompatibility complex, class II, DQ beta 1 [Source:HGNC Symbol;Acc:HGNC:4944]" | 2840 | 0.266 | 0.1600 | No |
| 12 | FAS | Fas cell surface death receptor [Source:HGNC Symbol;Acc:HGNC:11920] | 3828 | 0.173 | 0.0983 | No |
| 13 | INS | insulin [Source:HGNC Symbol;Acc:HGNC:6081] | 3885 | 0.166 | 0.1084 | No |
| 14 | FASLG | Fas ligand [Source:HGNC Symbol;Acc:HGNC:11936] | 3931 | 0.162 | 0.1189 | No |
| 15 | HLA-DOA | "major histocompatibility complex, class II, DO alpha [Source:HGNC Symbol;Acc:HGNC:4936]" | 4344 | 0.125 | 0.0977 | No |
| 16 | CD28 | CD28 molecule [Source:HGNC Symbol;Acc:HGNC:1653] | 4543 | 0.110 | 0.0918 | No |
| 17 | HLA-DPB1 | "major histocompatibility complex, class II, DP beta 1 [Source:HGNC Symbol;Acc:HGNC:4940]" | 5486 | 0.033 | 0.0215 | No |
| 18 | IL2 | interleukin 2 [Source:HGNC Symbol;Acc:HGNC:6001] | 5819 | 0.007 | -0.0037 | No |
| 19 | IL12B | interleukin 12B [Source:HGNC Symbol;Acc:HGNC:5970] | 5991 | -0.004 | -0.0167 | No |
| 20 | GZMB | granzyme B [Source:HGNC Symbol;Acc:HGNC:4709] | 6132 | -0.015 | -0.0262 | No |
| 21 | IFNG | interferon gamma [Source:HGNC Symbol;Acc:HGNC:5438] | 6410 | -0.036 | -0.0447 | No |
| 22 | HLA-DRA | "major histocompatibility complex, class II, DR alpha [Source:HGNC Symbol;Acc:HGNC:4947]" | 6550 | -0.046 | -0.0515 | No |
| 23 | CD80 | CD80 molecule [Source:HGNC Symbol;Acc:HGNC:1700] | 6918 | -0.074 | -0.0737 | No |
| 24 | IL12A | interleukin 12A [Source:HGNC Symbol;Acc:HGNC:5969] | 6930 | -0.074 | -0.0681 | No |
| 25 | HLA-DRB1 | "major histocompatibility complex, class II, DR beta 1 [Source:HGNC Symbol;Acc:HGNC:4948]" | 7087 | -0.086 | -0.0727 | No |
| 26 | HLA-F | "major histocompatibility complex, class I, F [Source:HGNC Symbol;Acc:HGNC:4963]" | 7196 | -0.096 | -0.0728 | No |
| 27 | IL1A | interleukin 1 alpha [Source:HGNC Symbol;Acc:HGNC:5991] | 7842 | -0.144 | -0.1104 | No |
| 28 | PRF1 | perforin 1 [Source:HGNC Symbol;Acc:HGNC:9360] | 8265 | -0.176 | -0.1279 | No |
| 29 | GAD2 | glutamate decarboxylase 2 [Source:HGNC Symbol;Acc:HGNC:4093] | 9249 | -0.252 | -0.1825 | No |
| 30 | HLA-DPA1 | "major histocompatibility complex, class II, DP alpha 1 [Source:HGNC Symbol;Acc:HGNC:4938]" | 9481 | -0.273 | -0.1768 | No |
| 31 | LTA | lymphotoxin alpha [Source:HGNC Symbol;Acc:HGNC:6709] | 9921 | -0.313 | -0.1837 | No |
| 32 | PTPRN | protein tyrosine phosphatase receptor type N [Source:HGNC Symbol;Acc:HGNC:9676] | 10712 | -0.395 | -0.2108 | Yes |
| 33 | HLA-G | "major histocompatibility complex, class I, G [Source:HGNC Symbol;Acc:HGNC:4964]" | 10869 | -0.413 | -0.1871 | Yes |
| 34 | HSPD1 | heat shock protein family D (Hsp60) member 1 [Source:HGNC Symbol;Acc:HGNC:5261] | 11427 | -0.492 | -0.1877 | Yes |
| 35 | HLA-E | "major histocompatibility complex, class I, E [Source:HGNC Symbol;Acc:HGNC:4962]" | 11659 | -0.526 | -0.1599 | Yes |
| 36 | IL1B | interleukin 1 beta [Source:HGNC Symbol;Acc:HGNC:5992] | 12105 | -0.622 | -0.1405 | Yes |
| 37 | HLA-A | "major histocompatibility complex, class I, A [Source:HGNC Symbol;Acc:HGNC:4931]" | 12502 | -0.749 | -0.1063 | Yes |
| 38 | HLA-B | "major histocompatibility complex, class I, B [Source:HGNC Symbol;Acc:HGNC:4932]" | 12534 | -0.764 | -0.0424 | Yes |
| 39 | HLA-DRB4 | "major histocompatibility complex, class II, DR beta 4 [Source:HGNC Symbol;Acc:HGNC:4952]" | 12636 | -0.815 | 0.0205 | Yes |
Table: GSEA details [plain text format]

  

Fig 2: KEGG\_TYPE\_I\_DIABETES\_MELLITUS      
 Blue-Pink O' Gram in the Space of the Analyzed GeneSet

  

Fig 3: KEGG\_TYPE\_I\_DIABETES\_MELLITUS: Random ES distribution      
 Gene set null distribution of ES for **KEGG\_TYPE\_I\_DIABETES\_MELLITUS**
